# Identification of chicken *FSHR* gene promoter and the correlations between polymorphisms and egg production in Chinese native hens

**DOI:** 10.1111/rda.13412

**Published:** 2019-03-23

**Authors:** Xiaopeng Li, Yinglin Lu, Xiaofan Liu, Xiaolei Xie, Kun Wang, Debing Yu

**Affiliations:** ^1^ Department of Animal Genetics, Breeding and Reproduction, College of Animal Science and Technology Nanjing Agricultural University Nanjing China

**Keywords:** association analysis, core promoter, *FSHR*, single nucleotide polymorphism

## Abstract

Egg production is an important economic trait in poultry, and it is of great significance to study the key genes and functional SNPs that affect egg laying performance. Follicle‐stimulating hormone (FSH) plays an important physiological role in the reproductive performance of humans and animals by binding to its receptor (FSHR). Studies have shown that there are many transcriptional regulatory elements in the 5′ flanking region of the *FSHR* gene that interact with transcription factors to regulate *FSHR* transcription. In this study, DNA sequencing was used to identify SNPs in the *FSHR* promoter sequence in both Dongxiang and Suken chickens. To detect the activity of the chicken *FSHR* gene promoter, we analysed the characteristics of the sequence and constructed three deletion vectors. We confirmed that the region (−18/−544) was the core promoter. Furthermore, five polymorphisms, including a 200‐bp indel at −869, C−1684T, C−1608T, G−368A and T−238A, were detected in both the Dongxiang and Suken chickens. The age at first egg (AFE) for different genotype of −869 indel in Suken chicken was significantly different (*p* < 0.01). For SNP C−1684T in Dongxiang chickens, the CC genotype had higher egg number at 43 weeks of age (E43) than that of the TC genotype (*p* < 0.05). For SNP C−1684T in Suken chickens, the TC genotype had higher AFE than that of the CC genotype (*p* < 0.05). For SNP C−1608T in Suken chickens, the CC genotype had higher AFE than that of the TC genotype (*p* < 0.05). For SNP G−368A in Suken chickens, the AG genotype had higher AFE than that of the GG genotype (*p* < 0.05).

## 1 INTRODUCTION

Dongxiang chicken is a kind of domestic chicken species in China, which produces eggs with blue shells (Wang, Liu, Wang, Li, & Deng, 2011). It is characterized by black feathers, black skin, black bones and black organs. The growth rate and egg yield of this variety are very low (Wang et al., 2009). Suken chicken is also a Chinese native breed, a kind of Chinese triple‐yellow chickens, which have yellow beak, yellow feather and yellow claw (Liu et al., 2014). Suken chicken has an egg production period of approximately 268 days throughout the egg laying cycle, and its egg production peak duration is approximately 40 days. In comparison, Dongxiang chicken has an egg production period of approximately 251 days throughout the egg laying cycle, and its egg production peak lasts for approximately 25 days.

Follicle‐stimulating hormone (FSH) is a glycoprotein “synthesized and secreted by the gonadotropic cells of the anterior pituitary gland (Pierce & Parsons, 1981)” that plays a vital role in gonadal function and fertility (George, Dille, & Heckert, 2011). After FSH is released into circulation, it plays a physiological function by binding to the specific transmembrane receptor (FSHR) located on the target cell (Heckert & Griswold, 1991). In 1996, the cDNA sequence of *FSHR* was first successfully cloned from chicken ovarian tissue (You, Bridgham, Foster, & Johnson, 1996). The sequence analysis and integrated results of the chicken *FSHR* gene were demonstrated in 2005 (Wicker et al., 2005). Studies have shown that *FSHR* is selectively expressed in Sertoli cells and ovarian granulosa cells (Camp, Rahal, & Mayo, 1991; Dankbar et al., 1995), and its expression level is closely related to germ cell differentiation and maturation (Heckert & Griswold, 1993). Gene promoters play a significant role in transcriptional regulation (Fan et al., 2016; Wang et al., 2015). At present, the *FSHR* promoter of humans, rats, mice and sheep has been successfully cloned (Gromoll, Dankbar, & Gudermann, 1994; Heckert, Daley, & Griswold, 1992; Levallet, Koskimies, Rahman, & Huhtaniemi, 2001; Sairam & Subbarayan, 1997), and the mechanism of transcriptional regulation has been extensively studied. Previous studies have shown that the promoter and transcription factors are closely related to promoter activity. For example, in the *FSHR* gene promoter of humans, rats, mice and sheep, the transcription factors USF1 and USF2 bind to the E‐box and regulate promoter activity (Goetz, Lloyd, & Griswold, 1996; Heckert, Daggett, & Chen, 1998; Hermann, Hornbaker, Rice, Sawadogo, & Heckert, 2008; Wood & Walker, 2009; Xing & Sairam, 2001). In addition, transcriptional regulators such as E2F, GATA1, SMAD3 and SF1 are also involved in the transcriptional regulation of the *FSHR* gene (Gong & McGee, 2009; Heckert, 2001; Kim & Griswold, 2001).

Although there are many studies on the regulation of mammalian *FSHR* promoters, the mechanism regulating transcription of chicken *FSHR* is not yet clear. In this study, the promoter region and sequence of the chicken *FSHR* gene were obtained by PCR and DNA sequencing. The transcription factor binding site (TFBS) of the chicken *FSHR* gene was predicted by online software, and the core promoter region was identified by a luciferase activity assay. Mice developed follicular dysplasia after *FSHR* gene knockout in granulosa cells (Kumar, Wang, Lu, & Matzuk, 1997). The *FSHR* gene promoter can regulate the transcription initiation site, time and expression level. Thus, in females, the SNPs occurring in the *FSHR* promoter region may affect the expression of the *FSHR* gene and influence reproductive performance. Researchers had found that the polymorphisms at the −278 site in the promoter region of the *FSHR* gene in Chinese Holstein cows significantly affected both the number of follicles and the number of transferred embryos (Yang et al., 2010).

Egg production is an important economic trait in poultry. Endocrine (Kim, Seo, & Ko, 2004) and many environmental factors (Lewis & Gous, 2006; Liu, Lilburn, Koyyeri, Anderson, & Bacon, 2004), such as photoperiod and different supplements, can affect egg laying performance. However, genetic factors play a decisive role in egg laying performance. Egg laying performance is controlled by multiple genes, and heritability is low. Furthermore, the laying performance of poultry in different periods is also very different (Emsley, 1997; Luo, Yang, & Yang, 2007). In poultry breeding, egg number at 43 weeks of age (E43) is usually an effective indicator of total egg production (Xu et al., 2011). There are obvious differences in egg laying performances of different breeds, including age at first egg (AFE), total egg number and egg weight. Because of the importance of the *FSHR* gene for reproductive performance, variations in *FSHR* gene expression may result in distinct reproductive performances in different chicken breeds. In addition, polymorphisms in the chicken *FSHR* gene promoter may also influence the transcription of *FSHR* and affect egg production in chickens. Accordingly, in this study, we detected nucleotide polymorphisms in the promoter of the *FSHR* gene of Dongxiang and Suken chickens by PCR‐RFLP. We then found that several polymorphisms among the five total polymorphisms were associated with E43 or AFE.

## 2 MATERIALS AND METHODS

Ethics Committee approval was obtained from the Institutional Ethics Committee of Nanjing Agricultural University to the commencement of the study.

### 2.1 Animals and DNA extraction

The chicken populations used for the experiment were Dongxiang (*n* = 116) and Suken chickens (*n* = 434) from Jiangsu Xincao Farm. The chicken was bred in cages (one chicken per cage) with the same feeding and management conditions. We recorded the age at first egg (AFE) and egg number at 43 weeks of age (E43) of every chicken.

We collected total 550 blood samples (116 for Dongxiang chicken and 434 for Suken chicken) from chicken wings and stored it at −20°C. The DNA was extracted by a conventional phenol–chloroform extraction method (Di Pietro, Ortenzi, Tilio, Concetti, & Napolioni, 2011) and adjusted to a final concentration of 100 ng/µl with ddH_2_O.

### 2.2 Primers

Ten pairs of primers shown in Table 1 were designed for the experiment. All primers were synthesized by JinWeiZhi Biotechnology Co., Ltd., China.

**Table 1:** Primers used for amplification of the follicle‐stimulating hormone receptor of Dongxiang and Suken chickens

Primer	Primer sequence	Tm/°C	Product size/bp	Application
P1	F:GGTATGGCTTACGCTTGTCTGT	62	790	Amplification
	R:GATTGTTTGCTTGTTTCTTTCG			
P2	F:AAAGGTGAGAATGGTGGAAT	59	553	Amplification
	R:CCAGAGCTAAATAACGCACC			
P3	F:AAAGGTGGTAGGGAGGAAGA	62	740	Amplification
	R:CCTGGCAGATGAATATCCTG			
P4	F:CGGggtaccACTCCCGTTCTTATGACACCTAT	61	1,461	Plasmids construction
	R:CCCaagcttTTGTCTCCTTCTCCTCCATC			
P5	F:CGGggtaccTTCTTGAACCTGTACCTCTTG	61	794	Plasmids construction
	R:CCCaagcttTTGTCTCCTTCTCCTCCATC			
P6	F:CGGggtaccTGGATCTATGAAGGGGAGC	61	526	Plasmids construction
	R:CCCaagcttTTGTCTCCTTCTCCTCCATC			
P7	F:GGTATGGCTTACGCTTGTCTGT	62	790	Genotyping
	R:GATTGTTTGCTTGTTTCTTTCG			
P8	F:TGTCTCTTAGTCTTATCAAACAACA	60	492	Genotyping
	R:CCTGGCAGATGAATATCCTG			
P9	F:AAAGGTGGTAGGGAGGAAGA	62	740	Genotyping
	R:CCTGGCAGATGAATATCCTG			
P10	F:ACAATCAAAACCCCAGCAAC	62	741	Genotyping
	R:AATGAACCGGAATGCTTTTG			

Note

The digestion sites of the enzymes are underlined.

### 2.3 PCR amplification and sequencing

PCR was performed in a 20 µl mixture containing 10 µl of 2X *Taq* Mix (Takara Biotechnology Co. Ltd., Dalian, China), 10 pmol of upstream and downstream primers and 100 ng of chicken genomic DNA. The following reaction conditions were used: 95°C pre‐denaturation for 5 min, 95°C denaturation for 30 s, X°C (X was the annealing temperature shown in Table 1) annealing for 30 s and a 72°C extension for 30 s (depending on product length, 1 kb = 1 min) for 35 cycles. The PCR products of P1, P2 and P3 were separated by 1.5% agarose gel electrophoresis and sequenced. SNPs were identified by sequence traces.

### 2.4 Analysis software

The promoter and transcription factor binding sites were predicted and analysed by Promoter Scan, Genomatix and Methprimer (Table 2).

**Table 2:** Software online for promoter analysis

Software name	URL	Purpose
UCSC	http://genome.ucsc.edu/	Promoter prediction
Promoter Scan	https://www-bimas.cit.nih.gov/molbio/proscan/	Core promoter prediction
Methprimer	http://www.urogene.org/methprimer/	CpG island prediction
Genomatix	http://www.genomatix.de/index.html	TFBS prediction

Note

TFBS: Transcription factor binding site.

### 2.5 Construction of the *FSHR* promoter luciferase plasmids

The purified promoter fragments of the chicken *FSHR* gene were amplified by three specific primers containing *KpnI* and *HindIII* restriction enzyme cleavage sites and then cloned into the pGL3‐basic vector digested with *KpnI* and *HindIII* restriction enzymes. The primers used to amplify the desired promoter fragments of the chicken *FSHR* gene are shown in Table 1.

### 2.6 Cell culture, transient transfection and luciferase activity assay

Specific methods for granulosa cell culture reference the article (Hu, Duggavathi, & Zadworny, 2017). The granulosa cells were seeded into 24‐well plates for 16–18 hr. The luciferase plasmids and the Renilla luciferase reporter vector (pRL‐K) were cotransfected at a ratio of 50:1 into chicken ovarian granulosa cells with Lipofectamine 2000 when the cells were completely adhered. The cells were collected after 24 hr of transfection, and luciferase activity was assayed using the Dual‐Luciferase® Reporter Assay System (Promega, Madison, WI, USA).

### 2.7 Genotyping of polymorphisms

Using the PCR‐RFLP method to detect genotypes, we selected the appropriate endonuclease for enzyme digestion of the target genes of the tested chickens. Enzyme reaction conditions followed the enzyme product specifications. Genotypes were detected by the gel bands graphic of 1.5% agarose gel electrophoresis. Electrophoresis conditions included the following specifications: 1X TBE, 120 V/30 min.

### 2.8 Statistical analysis

Allele and genotype frequencies were calculated by direct counting. The chi‐squared test was used to examine the Hardy–Weinberg equilibrium of the SNPs. Association analyses of SNPs with E43 and AFE were performed using spss version 20.0.

## 3 RESULTS

### 3.1 Chicken *FSHR* Gene 5′ regulatory region amplification

**Figure 1:**
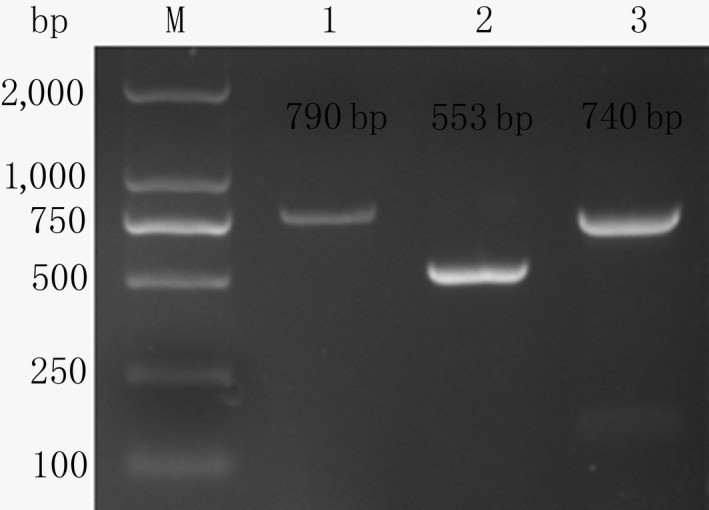
Agarose gel photograph of 5′ regulatory region of Chicken *FSHR* gene. 1‐3: Amplified fragments of primers P1‐P3; M: DNA marker DL2000

Three fragments of approximately 790 bp, 553 bp and 740 bp were separated in 1.5% agarose gel, after PCR amplification (Figure 1).

### 3.2 5′ regulatory sequence analysis of the chicken *FSHR* gene

**Figure 2:**
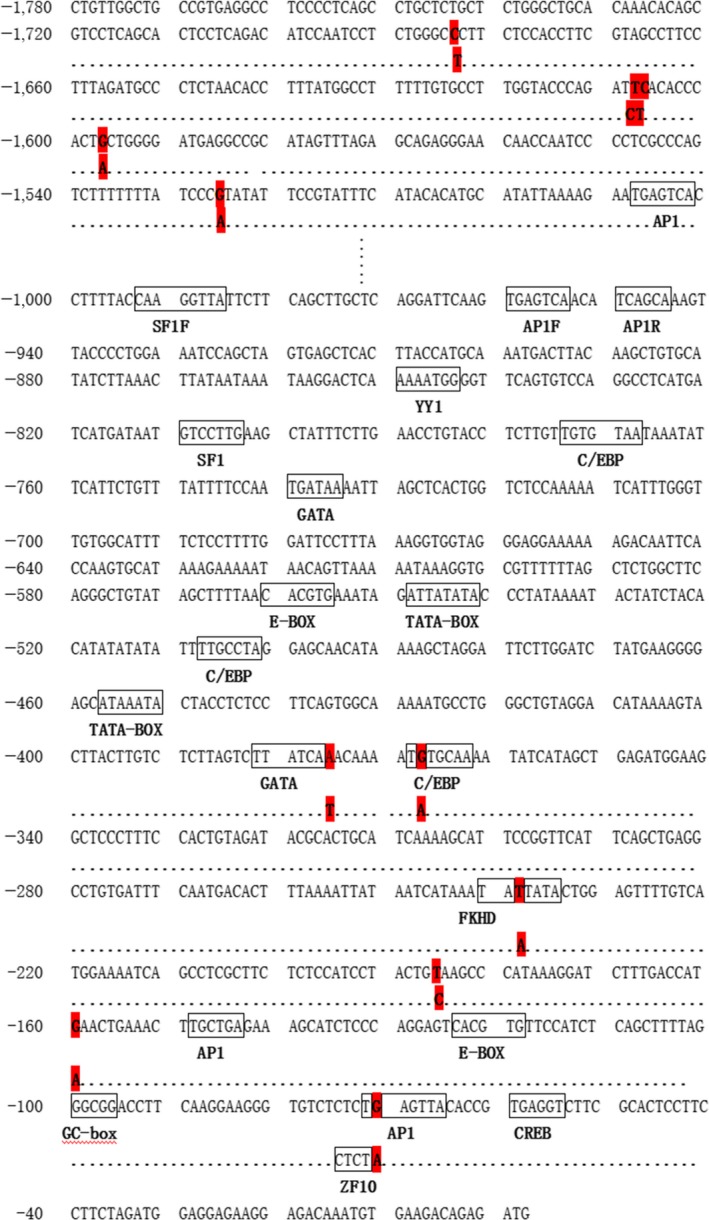
The 5′ regulation sequence of *FSHR* gene in chicken. The SNP sites are indicated by red background; the transcription factor binding sites are indicated by blue boxes

**Figure 3:**
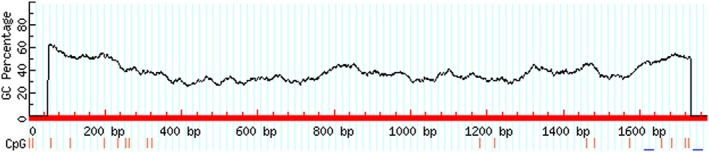
The prediction result of CpG islands in promoter region of Chicken *FSHR* gene. Vertical lines indicate CpG sites

A few common cis‐elements were predicted in the chicken *FSHR* proximal promoter sequence by Genomatix online software (http://www.genomatix.de/index.html), including two TATA‐boxes (TBP binding site), three CAAT‐boxes (C/EBP binding sites), a GC‐box (SP1 binding site) and two E‐boxes (USF1/2 binding sites). In addition, several transcription factor binding sites were enriched in the chicken *FSHR* proximal promoter sequence, including AP1, SF1, YY1, GATA, FKHD, SP1 and CREB (Figure 2). No typical CpG islands were detected in the chicken *FSHR* proximal promoter sequence using Methprimer online software (http://www.urogene.org/methprimer/; Figure 3).

### 3.3 Promoter activity analysis of the chicken *FSHR* gene

**Figure 4:**
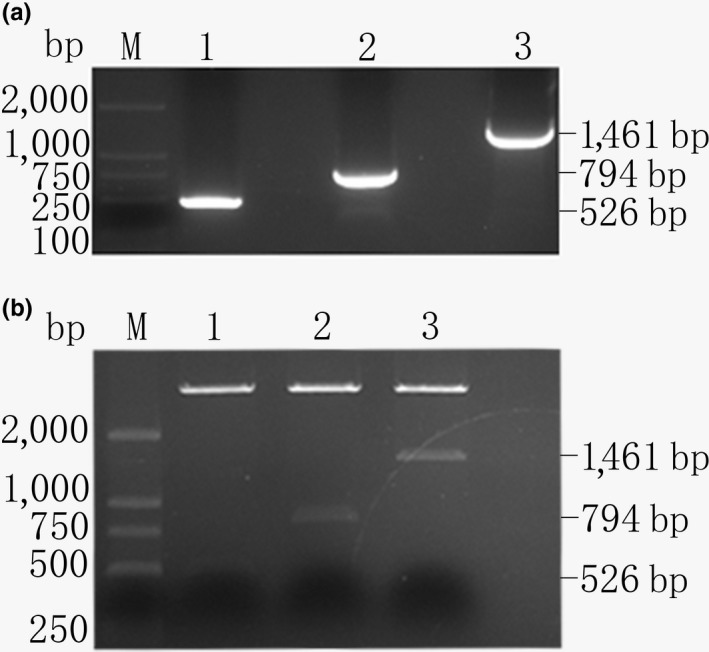
A agarose gel photograph of deleted fragment in 5′ regulatory region of Chicken *FSHR* gene. B identification of recombinant vectors by restriction enzymes

**Figure 5:**
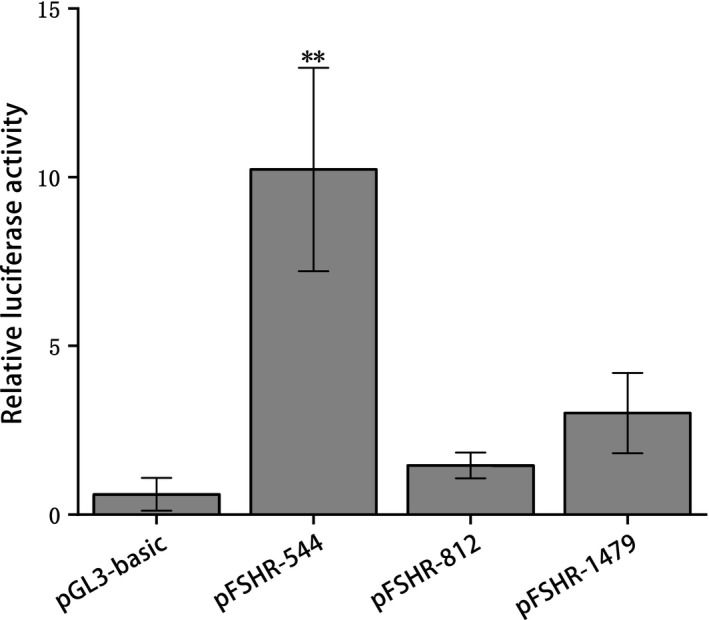
Promoter activity analysis of Chicken *FSHR* gene. ** indicates extremely significant difference (*p* < 0.01)

PCR product electrophoresis is shown in Figure 4a. Three special plasmids, p*FSHR*‐1479, p*FSHR*‐812 and p*FSHR*‐544, were constructed to identify the promoter activity of the chicken *FSHR* gene, and the translation start site (ATG) was defined as +1. The constructed plasmids were identified by double digests (Figure 4b). The plasmids were transiently transfected into chicken ovarian granulosa cells, and luciferase activity assays were performed to identify the promoter activity of the chicken *FSHR* gene. As shown in Figure 5, the luciferase activity of the promoter p*FSHR*‐544 was significantly higher than that of p*FSHR*‐812, p*FSHR*‐1479 and the negative control pGL3‐basic (*p* < 0.01). In contrast, no significant difference was observed between p*FSHR*‐1479, p*FSHR*‐812 and pGL3‐basic (*p* > 0.05).

### 3.4 Genotype frequency and allele frequency

**Figure 6:**
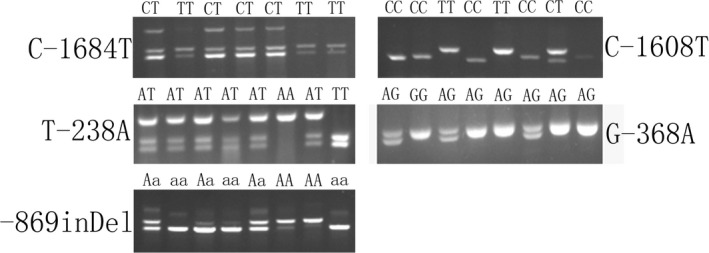
Genotyping of the C−1684T, C−1608T, T−238A, G−368A and −869 indel mutations of the promoter region of chicken *FSHR* gene

We found that four restriction sites exist at C−1684T, C−1608T, G−368A and T−238A within the promoter region of chicken *FSHR*, including *ApaI*, *MboI*, *NdeI* and *SspI*, respectively. The four single nucleotide polymorphisms and the 200‐bp indel mutation were detected by PCR‐RFLP (Figure 6).

All five mutations were in a Hardy–Weinberg imbalanced state in the Suken yellow chicken population. Furthermore, the −869 indel and G−368A mutations were in a Hardy–Weinberg imbalanced state in the Dongxiang chicken population (Table 3).

**Table 3:** Genotypes and allele frequency of the mutations of *FSHR* gene

Polymorphism sites	Breed	Genotypic frequency			Allele and frequency		*χ* ^2^
		Genotype	Number	Frequency	Allele	Frequency	
−869 indel	Dongxiang	AA	4	0.04	A	0.082	15.830*
		Aa	11	0.09	a	0.918	
		aa	101	0.87			
	Suken	AA	10	0.02	A	0.089	15.29*
		Aa	57	0.13	a	0.911	
		aa	367	0.85			
C−1684T	Dongxiang	CC	42	0.36	T	0.379	1.125
		TC	60	0.52	C	0.621	
		TT	14	0.12			
	Suken	CC	85	0.30	T	0.518	9.051*
		TC	248	0.53	C	0.482	
		TT	101	0.17			
C−1608T	Dongxiang	CC	39	0.34	T	0.457	3.204
		TC	48	0.41	C	0.543	
		TT	29	0.25			
	Suken	CC	312	0.72	T	0.217	166.7*
		TC	56	0.13	C	0.783	
		TT	66	0.15			
G−368A	Dongxiang	GG	73	0.63	A	0.185	6.004*
		AG	43	0.37	G	0.815	
		AA	0	0			
	Suken	GG	252	0.58	A	0.210	30.55*
		AG	182	0.42	G	0.790	
		AA	0	0			
T−238A	Dongxiang	AA	63	0.54	A	0.720	1.775
		AT	41	0.35	T	0.280	
		TT	12	0.11			
	Suken	AA	138	0.32	A	0.530	9.64*
		AT	184	0.42	T	0.470	
		TT	112	0.26			

*χ* ^2^_0.05 (2)_ = 5.99, *χ* ^2^_0.05(1)_ = 3.84, *χ* ^2^_0.01(1)_ = 6.63.

The chi‐square value with * means *p* < 0.05.

### 3.5 Association analysis of SNPs of the chicken *FSHR* gene with E43 and AFE

The association analyses of SNPs with E43 and AFE was performed using SPSS version 20.0 (Tables 4 and 5).

**Table 4:** Association analysis of SNPs of *FSHR* gene with Dongxiang chicken egg performance

Polymorphism sites	Genotype	Number	AFE	*p*‐Value	E43	*p*‐Value
−869 indel	AA	4	170.00 ± 7.53	0.704	56.25 ± 6.95	0.304
	Aa	11	165.45 ± 10.21		60.82 ± 15.03	
	aa	101	165.28 ± 16.33		66.37 ± 16.93	
C−1684T	TT	14	168.00 ± 14.82	0.222	65.71 ± 12.78 ^ab^	0.050
	TC	60	167.28 ± 19.21		62.77 ± 19.11 ^b^	
	CC	42	162.19 ± 8.08		69.33 ± 13.08 ^a^	
C−1608T	TT	29	165.14 ± 10.24	0.982	62.76 ± 19.20	0.287
	TC	48	165.48 ± 19.85		68.35 ± 16.27	
	CC	39	165.87 ± 13.19		64.03 ± 14.76	
G−368A	GG	73	165.89 ± 17.19	0.465	65.85 ± 16.07	0.569
	AG	43	164.91 ± 12.70		64.91 ± 17.67	
	AA	0	0		0	
T−238A	TT	12	164.08 ± 11.57	0.800	72.42 ± 13.70	0.292
	AT	41	164.59 ± 16.44		65.51 ± 14.01	
	AA	63	166.41 ± 15.90		64.17 ± 18.46	

Note

In the same group, different superscripts mean significant difference (*p* < 0.05).

**Table 5:** Association analysis of SNPs of *FSHR* gene with Suken chicken egg performance

Polymorphism sites	Genotype	Number	AFE	*p*‐Value	E43	*p*‐Value
−869 indel	AA	10	160.40 ± 5.54^A^	0.005	96.70 ± 19.33	0.390
	Aa	57	157.25 ± 2.90^B^		104.19 ± 14.27	
	aa	367	157.12 ± 3.06^B^		103.40 ± 16.18	
C−1684T	TT	101	157.24 ± 3.14^ab^	0.030	101.70 ± 16.35	0.452
	TC	248	157.48 ± 3.70^a^		104.08 ± 15.85	
	CC	85	156.58 ± 2.09^b^		103.18 ± 16.12	
C−1608T	TT	66	156.74 ± 3.60^ab^	0.023	102.67 ± 17.30	0.234
	TC	56	156.35 ± 1.92^b^		106.75 ± 16.45	
	CC	312	157.51 ± 3.42^a^		102.88 ± 15.63	
G−368A	GG	252	156.83 ± 2.88^b^	0.003	104.27 ± 15.27	0.161
	AG	182	157.83 ± 3.78^a^		102.08 ± 16.95	
	AA	0	0		0	
T−238A	TT	112	157.08 ± 3.29	0.674	104.19 ± 16.99	0.758
	AT	184	157.20 ± 3.20		103.35 ± 15.45	
	AA	138	157.44 ± 3.52		102.67 ± 16.02	

Note

In the same group, different superscripts mean significant difference (*p* < 0.05).

Dongxiang chickens with the CC genotype of SNP C−1684T had higher E43 compared with that of chicken with the TC genotype (*p* < 0.05) (Table 4). The AFEs of the SNP −869 indel and SNP G−368A genotypes were significantly different (*p* < 0.01). The AFEs of the SNP C−1684T and C−1608T genotypes were also significantly different (*p* < 0.05; Table 5).

## 4 DISCUSSION

Gene promoters play a key role in transcriptional regulation by controlling the transcription initiation site, time and expression level (Juneja, Ilm, Schlag, & Stein, 2013). Therefore, research on the regulation of gene expression can start from the structural function of its promoter. In this study, we obtained approximately 1.8 kb sequence of the chicken *FSHR* gene promoter region and analysed the structure using bioinformatics software, revealing predicted TATA‐box and CAAT‐box cis‐acting elements. The TATA‐box and CAAT‐box are not found in the promoter region of the human, rat and sheep *FSHR* genes. However, there is a TATA‐box in the mouse *FSHR* gene promoter region (Gromoll et al., 1994; Heckert et al., 1992; Sairam & Subbarayan, 1997), which indicating that the regulatory mechanism of the FSHR gene promoter may differ interspecifically.

A recently research indicated that miR‐4281, an miRNA specifically expressed in hominids, directly interacting with the TATA‐box motif in the human *FOXP3* promoter could efficiently and specifically upregulates *FOXP3* expression (Zhang et al., 2018)**. **Overexpression of CAAT/enhancer‐binding protein (C/EBP) in Spodoptera litura‐221 (Spli‐221) cells increased the promoter activity 5.57‐fold, while mutation of the C/EBP CRE abolished the binding of the C/EBP with the CRE (Liang, Zhang, Zeng, Zheng, & Feng, 2015). These findings validate the important role of TATA‐box and CAAT‐box in promoter regulation.

The E‐box was mutated in the promoter of the rat *FSHR* gene, which resulted in a significant reduction of *FSHR* promoter activity in MSC‐1 cell lines (Heckert et al., 1998). Two E‐box sites were predicted to be present in the promoter of the chicken *FSHR* gene, whether the E‐box regulating the *FSHR* promoter requires further identification. Furthermore, multiple transcription factor binding sites (TFBS) were predicted in the chicken *FSHR* gene promoter with Genomatix software, including AP1, GATA, SF1, YY1, as well as others. It is noteworthy that the transcription factors E2F, Smad3 and ETS were involved in the transcriptional regulation of the *FSHR* gene in previous reports (Brune, Adams, & Gromoll, 2010; Heckert, 2001; Kim & Griswold, 2001). However, we did not predict these transcription factor binding sites in the chicken *FSHR* gene promoter, further illustrating that different promoter regulatory mechanisms are likely to exist in the *FSHR* genes in different species.

Researchers have identified that the region (−1,195/−598) was the core promoter of the porcine *FSHR* gene (Wu et al., 2015). In order to identify the core promoter region of the chicken *FSHR* gene, we constructed three special plasmids: p*FSHR*‐1479 (−1,479/−18), p*FSHR*‐812 (−830/−18) and p*FSHR*‐544 (−562/−18). The activity of p*FSHR*‐544 was significantly higher than that of p*FSHR*‐basic, p*FSHR*‐1479 and p*FSHR*‐812. There is no significant difference in the activity between p*FSHR*‐1479 and p*FSHR*‐812 and the p*FSHR*‐basic. The above studies suggested that the region (−18/−562) contains some positive cis‐regulatory elements, whereas the region (−562/−1,497) contains some negative transcription factor binding sites.

The sequencing results showed that eleven SNPs exist in the promoter of the chicken *FSHR* gene. It has been widely reported that mutations in the *FSHR* gene have a genetic effect on reproductive traits in humans and other animals (Lussiana et al., 2008). Researchers had found that the *FSHR* promoter polymorphism *FSHR *−29G>A influences the androgen levels of human small antral follicle (hSAF; Borgbo et al., 2017). The activity of *FSHR* promoter is significantly affected by the 29th site G → A mutation that will weaken promoter activity and result in poor response to FSH (Dan, Jing, Liangbin, Ting, & Ying, 2015). We analysed the effect of *FSHR* gene polymorphism on transcription factor binding sites and found that there are three mutations leading to changes in the transcription factor binding site. However, it remains to be investigated whether the changes of these transcription factor binding sites will have an effect on the transcriptional activity of chicken *FSHR*.

Furthermore, the genotypes of five SNPs were associated with both E43 and AFE. The SNP C−1684T of Dongxiang chicken was associated with E43 and SNPs C−1684T, C−1608T and G−368A of Suken chickens were significantly related with AFE. Moreover, in this study, the 200‐bp indel mutation had a significant correlation with AFE, which was compatible with the findings of Kang et al. (2012). Our data suggested that these loci might serve as the potential genetic markers for chicken reproduction. Previous study of *FSHR* gene in muscovy duck detected that the SNP C320T is significantly associated with egg production at 59 weeks of age (*p* < 0.05), whereas the SNP A227G is significantly associated with age at first egg stage (*p* < 0.05) (Xu et al., 2017). However, the mechanisms by which these polymorphisms make their effects require to be further researched.

In conclusion, in this study, we identified the core promoter region of the chicken *FSHR* gene and predicted several transcription factor binding sites. Moreover, a total of five polymorphisms of the *F*SHR promoter region were detected, and we found that all of them were associated with egg number at 43 weeks of age (E43) or age at first egg (AFE).

## CONFLICT OF INTEREST

None of the authors have any conflict of interest to declare.

## AUTHOR CONTRIBUTIONS

Debing Yu and Yinglin Lu designed the experiment. Xiaopeng Li, Xiaofan Liu, Xiaolei Xie and Kun Wang completed the experiment. Xiaopeng Li wrote and revised the paper.
